# Cross-class interactions and subjective inequality: perceptions, beliefs and distributive preferences at a Colombian elite university

**DOI:** 10.3389/fsoc.2025.1619937

**Published:** 2025-09-16

**Authors:** Andres Mauricio Galeano-Salgado, María José Álvarez-Rivadulla

**Affiliations:** Faculty of Social Sciences. University of Los Andes, Bogotá, Colombia

**Keywords:** cross-class interactions, inequality perceptions, inequality beliefs, distributive preferences, contact hypothesis, higher education

## Abstract

This study examines how cross-class interactions influence perceptions and beliefs of inequality, and distributive preferences. It is based on the implementation of *Ser Pilo Paga*, a government program that granted access to high-quality higher education for low-income students in Colombia. Drawing on 61 in-depth interviews and complementary survey data, we find that exposure to peers from different socioeconomic backgrounds recalibrates students’ understanding of inequality, making their perceptions more accurate and their attitudes toward redistribution more favorable—regardless of class. While students consistently held structural views of inequality, these interactions reshaped their views on merit, revealing its dual function as both a source of validation and a relational tool. Notably, we find that meritocratic beliefs coexisted with structural critiques, challenging assumptions that meritocracy legitimizes inequality. Finally, support for the fellowship program was nuanced and ambivalent, particularly among beneficiaries, who recognized both its benefits and its limitations as a redistributive mechanism. These findings advance sociological understandings of subjective inequality by highlighting how class contact in segregated societies can shift beliefs and preferences in ways that challenge dominant theories of self-interest and merit-based legitimation.

## Introduction

Social segregation is a widely extended phenomenon along different domains of social life in contemporary societies. From transport to housing and education, segregation has produced societies in which people inhabit worlds that are different to an extent that it is difficult for individuals to gauge the actual levels of inequality that exist around them (Mijs and Usmani, 2024). Segregation actively excludes the most disadvantaged groups of population and promotes the closure of spaces where a small number of privileged individuals access and hoard opportunities, thus reproducing inequality and poverty (Kaztman, 2007; Desmond, 2023). Social integration, meanwhile, is often proposed as a mechanism to change inequality perceptions and challenge prejudices (Allport, 1954), especially in highly segregated societies. If segregation under high inequality settings hinders an accurate perception of inequality, social integration might transform individuals’ inequality perceptions and drive more redistributive demands with respect to resources and opportunities. This is precisely the hypothesis that this paper tackles for a specific context of class integration in an educational setting.

Research across the social sciences has found inequality to be generally misperceived due to a variety of factors. Objective levels of inequality are not necessarily consistent with the levels that people grasp (Trump, 2023a) and these perceived levels are related to meritocratic beliefs instead (Mijs, 2021). The perception of oneself in relation to others below or above in the income scale may also change how inequality is questioned or legitimized (Sherman, 2019). Moreover, segregation also reduces the possibility of perceiving inequality correctly because in segregated societies, networks are formed under patterned formative institutions—as the family, school or neighborhood—leading people to learn about inequality from a sample that does not give them enough information to infer inequality levels correctly (Mijs and Usmani, 2024). Nevertheless, diversifying one’s social networks and discussing political issues related to income distribution has been shown to make people more aware of inequality and more supportive of redistribution (Newman, 2014; Sumaktoyo et al., 2022). This shows that perceptions of inequality and distributive attitudes may change over time depending on one’s social environment and networks composition and characteristics.

In such segregated societies, educational experiments in the form of vouchers, racial quotas, magnet schools and so forth, are one of the few contexts where we can see the effect of cross class interactions on perceptions and attitudes toward inequality. Qualitative research has shown how students from different social classes interact among them and with other members of the school or college community, displaying their attitudes toward others’ social status and inequality more broadly (Stuber, 2006, 2011). Students exhibit different forms of adaptation to the context of college ranging from camouflaging their actual class position to disclosing it (Álvarez Rivadulla, 2014; Álvarez–Rivadulla et al., 2023) or changing the way they interact with people from different social classes, such as service personnel at school (Khan, 2011). A more quantitative branch of research has shown that the sole fact of sharing classes with students from other social sectors makes inequality perceptions more accurate and may turn the more privileged individuals more prone to altruistic or pro-social attitudes and behaviors (Londoño-Vélez, 2022).

To contribute to the understanding of subjective experiences of inequality, this study brings together literature on social contact, inequality perceptions and distributive attitudes and builds on the case of a government program to promote access to high-quality college education for low-income and academically outstanding students in Colombia. Between 2015 and 2018, the Ser Pilo Paga program benefited around 40.000 students and changed the social composition of universities in this country, promoting social encounters that would have been unlikely otherwise (Álvarez-Rivadulla, 2024b). We delve into the context of Study University, a high-quality elite college in Bogotá, the capital city of Colombia, to explore the following question: how do inequality beliefs and distributive preferences vary by social class in a context of unusual or exceptional cross-class interactions? Drawing primarily on the analysis of 61 in-depth interviews with students from different social classes and on secondary quantitative data analysis from a two-wave survey to students from four different majors, we develop some mechanisms that explain how the relationship among cross-class interactions, inequality perceptions and beliefs, and distributive preferences unfolds.

Students from different social classes held structural beliefs about inequality, viewing it as the result of social forces beyond individual control rather than personal effort and talent (Kluegel and Smith, 1986). However, we gained further understanding of how cross-class interactions led students to recalibrate their inequality perceptions, making them more accurate and often associating this with stronger support for redistribution, regardless of their social class, which challenges our understanding of the importance of self-interest for shaping distributive preferences. At the same time, we show how structural inequality beliefs are not incompatible with different comprehensions and valuations of merit in students’ understanding, thus challenging current assumptions in academic literature regarding meritocratic beliefs as legitimizers in unequal societies (Mijs, 2021). In our educational context, merit serves not only as a potential equalizer but also as a catalyst for social connections, at the same time that it reminds everybody that it is not enough to reach mobility or equality. While the poor see that many close others with similar merit did not make it, the rich see that they are in the University perhaps with less merit or at least less effort than their peers. Finally, we found ambivalent views of the Ser Pilo Paga program among students, especially among its recipients. This, contradictory again with the self-interest hypothesis, makes complete sense in their intensified structural perspective. Particularly among beneficiaries, and beyond other specific criticisms to the program implementation, there is a growing awareness that while the program may alter their individual trajectories, it fails to address broader inequalities by excluding other equally deserving individuals. This disjuncture reveals for them the limitations of meritocratic interventions in the absence of deeper structural reform. In sum, we provide new evidence and mechanisms to understand how cross-class interactions can transform inequality perceptions, beliefs, and distributive preferences.

### Subjective inequality: perceptions, beliefs, tolerance and preferences

The study of inequality has gone far beyond the understanding of unequal distributions of income, goods or opportunities—what some call objective inequality. An important branch of research has been dedicated to the study of subjective inequality, which relates to the perceptions, beliefs and judgments about those unequal distributions (Janmaat, 2013). The relevance of subjective inequality for sociological research lies in its potential to shed light on the ways in which inequality is interpreted by individuals and how, under different conditions, these interpretations can lead to reproducing or questioning inequalities (Bottero, 2020). Thinking through these lenses, this study focuses on the influence of cross-class interactions over different but interrelated aspects of subjective inequality: inequality perceptions, beliefs, tolerance to inequality, and distributive preferences.

On the one hand, perceptions of inequality are subjective estimates of how unequal a society is. When forming inequality perceptions “people make estimates of the magnitude of inequality and about the extent to which the existing distribution of income is based on merit, equality or ascription” (Janmaat, 2013, p. 359). Both quantitative and qualitative research have found that income inequality is generally misperceived by individuals (Gimpelson and Treisman, 2018), a trend driven by factors as systematic biases stemming from individuals’ statistical inference problem, social sampling, flaws in survey research design and different types of orientation to others.

The individuals’ statistical inference problem refers to the fact that people perceive inequality based on the income levels that they observe among a sub-sample of the population, which gives limited information to estimate the overall levels of inequality (Cruces et al., 2013; Mijs and Usmani, 2024). This argument relates closely to cognitive explanations of perceptions coming from the social sampling approach, which suggests that individuals’ rely on their closest social circles and environments to estimate broader characteristics in the rest of the population (Galesic et al., 2012). In addition, flaws in survey research design refer to difficulties to account correctly for inequality perceptions using certain survey items. Specifically, Trump (2023b) highlights that traditional survey items used to measure inequality perceptions require a level of numeric ability and awareness that respondents usually do not have, leading to inaccurate responses.

Qualitative research has also shown how misperception of inequality is constructed through everyday interactions and comparisons. For example, Michèle Lamont (1994, 2000) studied how upper-middle class and working-class individuals in the United States and France construct different meanings about one another through the creation of moral and socioeconomic boundaries which are closely linked to the daily and subjective experience of inequality. Similarly, Rachel Sherman’s work on New York elite families explored how they tend to think of themselves as middle-class regardless of their income. This occurs due to upward orientations to others, which is thinking of oneself position in the income distribution by comparing with those above own status, but not with those below (Sherman, 2019).

Beyond measurement issues, several studies have also examined factors influencing perceptions of inequality. Some of them have found that actual levels of income inequality are not related to subjective perceptions (Trump, 2023a), while factors like meritocracy (Mijs, 2021), class position (Knell and Stix, 2020; Haddon and Wu, 2022) and social mobility (Gimpelson and Monusova, 2014) may affect the way people perceive inequality. In the specific context of Colombia, García-Sánchez et al. (2018) used an online survey and found that Colombians frame inequality not only in terms of income, but also around aspects like opportunities in education, access to public services and spaces, and cross-class relations. Our study aims to build on these findings by exploring how cross-class interactions can lead to more accurate perceptions of inequality in segregated societies through mechanisms like the recalibration of inequality, as we will discuss below.

While perceptions concern how much inequality exists, beliefs explain *why* it exists. Inequality beliefs typically fall on a spectrum that ranges from individualistic or meritocratic beliefs to structuralist beliefs (Kluegel and Smith, 1986). The literature has defined these views as follows: the individualistic or meritocratic belief holds that individuals’ effort and talent are the cause of their position in society, while the structuralist belief places the cause on society and broader structures, suggesting that individual outcomes are the product of a set of conditions that people cannot control and that foster or hinder their possibilities to succeed or fail, like class, skin color, family resources, among others (Kluegel and Smith, 1986; Mijs, 2018). Social mobility has been examined as a factor that influences these beliefs, as in the work by Mijs et al. (2022) in the Netherlands which showed that those who experienced upward social mobility had stronger meritocratic beliefs. Moreover, perceptions of inequality and inequality beliefs interact and produce broader attitudes toward inequality. For example, someone may perceive high inequality and justify it on the grounds of merit or question it because of a structural view, reflecting different levels of tolerance to inequality.

Previous research in the Latin American context has suggested that sociological research should inquire about the levels of tolerance to inequality to understand social relations and cohesion in unequal contexts (Kaztman, 2007). Tolerance to inequality refers to attitudes and behaviors that question or legitimize inequalities. Attitudes include distributive preferences or the questioning or naturalization of the existing distribution, while behaviors entail how different ascribed factors—like class, race or gender—take a role in the unfolding of interactions among groups with different social positions (Álvarez Rivadulla, 2014). Thus, higher levels of tolerance to inequality may be reflected in lower support for redistribution and lower tolerance in stronger redistributive demands. However, people can simultaneously question and legitimize inequality along different domains or depending on the context (Álvarez Rivadulla, 2014) leading to what the scholarship has named a “split consciousness,” in which egalitarian and individualistic beliefs of inequality coexist (Castillo, 2011). Drawing on these concepts, we examine how cross-class interactions may modify the way inequality perceptions and beliefs together produce different results in terms of tolerance to inequality.

Distributive preferences, one of the attitudinal components of tolerance to inequality, are individual beliefs about how income distribution should be, including ideas about the redistributive role of the state, the amount of income that should be redistributed and the best mechanisms for doing it (Alesina and Giuliano, 2011). Moreover, distributive preferences are composed by at least two facets, as defined by Cavaillé and Trump (2015): the evaluation of being a potential beneficiary of redistribution *from* the rich or a potential contributor to redistribution *to* the poor. Simultaneously, these evaluations are connected to fairness reasoning, “the thought process through which individuals act as if a third-party judge ruling on the fairness of a given situation and acting to maximize fairness accordingly” (Cavaillé, 2023, p. 11). Fairness reasoning is structured by the proportionality belief and the reciprocity belief. The former holds that individuals should be rewarded based on merit, understood as the combination of decisions and talent, while the latter emphasizes the importance of cooperative behavior and social solidarity (Cavaillé, 2023).

Research across the social sciences has tried to explain distributive preferences, leading to the prevalence of two competing explanations: self-interest and altruism. The self-interest explanation comes from the seminal work of Meltzer and Richard about distributive preferences (Meltzer and Richard, 1981). There, the authors propose a model that suggests that support for redistribution comes from welfare-maximizing choices by individuals that aim to maintain or improve their position in case the distribution changes. On the contrary, the rival explanation of altruism sustains that concerns about social welfare and about the utility of others are important determinants of distributive preferences (Alesina and Giuliano, 2011; Dimick et al., 2018). Class identity has also been analyzed as an explanation of distributive preferences, although its importance varies considerably across contexts: in the United States it is not significant when controlling for other variables, while in Scandinavian countries both objective and subjective class position configure these preferences (Lindh and McCall, 2020). For the Latin American case, there is a wide support for redistribution by the state, but it has decreased over the years (Franetovic and Castillo, 2022). Furthermore, in this region there is mixed evidence about determinants of distributive preferences. Álvarez-Rivadulla (2024b), for example, found that both self-interest and altruism shape distributive preferences in Costa Rica and Uruguay, but did not have the same relevance in Chile and Colombia. Finally, research about distributive preferences in Colombia also shows that there are, at least, two components of these preferences: the one focused on taxing the wealthy and the one focused on helping those in need (García-Sánchez et al., 2022). In this context, support for redistribution is also shaped by the experiences of exclusion from welfare benefits which leads to diminished expectations toward redistribution, reducing support for it (Holland, 2018). The case analyzed in this study brings cross-class interaction as another factor that shapes distributive preferences—and subjective experiences of inequality more broadly—in highly unequal societies and sheds light on various mechanisms that explain this process.

### Cross-class interactions and subjective inequality

Another body of research to which this study contributes is the one on cross-class interactions. Gordon Allport’s classical work on prejudice proposed the so-called contact hypothesis, which points out that contact between groups in hierarchical societies can help to reduce prejudice (Allport, 1954). A meta-analysis by Pettigrew and Tropp (2006) found that intergroup contact effectively reduces prejudice and that it works not only among racial groups, but also among other social groups. This suggests that intergroup interactions can be beneficial and change the way in which groups perceive each other. Besides reducing prejudice, intergroup contact can influence distributive preferences. For instance, Sumaktoyo et al. (2022) found that social contacts’ perceived income changes preferences for redistribution depending on class position in Germany. Similarly, Newman (2014) showed that having friends that struggle financially raises awareness about inequality and unjust income distribution in the United States. In Latin America, Otero and Mendoza (2023) showed very similar effects for the Chilean context, where socioeconomically diverse networks are associated with greater consciousness about inequality and more egalitarian distributive preferences.

Several research has been done in the context of higher education to understand the dynamics and impacts of cross-class interactions. For example, Shamus Khan’s ethnography, *Privilege*, about an elite school in the United States shows that upper-class students develop closer relationships with service personnel at the school while middle-class students make a greater effort to differentiate (Khan, 2011). At the same time, cross-class interactions are important to understand how people perceive inequality in this context because they drive processes of meaning construction around inequality and class. The ethnographic work by Stuber (2006) is an example of this as she shows how contact with peers from diverse socioeconomic backgrounds shapes perceptions about social classes and inequality more broadly. Similarly, evidence from an affirmative action policy in Indian elite schools showed that social integration promoted prosocial behavior among upper-class students (Rao, 2019). In terms of redistributive support, cross-class interactions may also foster what some scholars have called “parochial altruism,” which is “altruism bounded by perceptions of common group membership or shared experience” (Lupu and Pontusson, 2011, p. 318), which is likely to happen in contexts like college. For instance, Jonathan Mijs (2023) found that interactions with peers from different socioeconomic and racial backgrounds shape inequality beliefs in American colleges, often attenuating meritocratic views when students have roommates from other SES and races. This study contributes to the field with evidence from a rare policy experiment in another context with high class-segregation in education settlements, the Colombian one. It proposes comparing elite and non-elite students rather than focusing on one of these groups, which has rarely been done before.^1^

### The case: social interactions in a segregated context

Colombia is widely known as one of the most unequal countries in the world and in Latin America. Despite considerable economic growth in the region thanks to the commodity boom during the first decades of the 21^st^ century, Colombia was one of the countries with the lowest rates of inequality reduction (Benza and Kessler, 2020). Education is one of the most class-segregated contexts in the country, where private education offers quality and opportunities for the most privileged who can afford it, while the less privileged usually access public education of lower quality. This is transmitted intergenerationally and has created an educational apartheid (García Villegas et al., 2021; García et al., 2015). The upper-middle class prefers private schools since they not only offer quality but also are a sign of social status (Álvarez Rivadulla, 2024a). Moreover, the meaning of middle classness is deeply rooted in private education, in contrast to other contexts in the region where middle classness is associated to public education like Argentina or Uruguay (Álvarez-Rivadulla, 2023). This segregation occurs from elementary school and is reproduced to secondary and post-secondary education (Fergusson and Flórez, 2021). Thereby, cross-class interactions are very unlikely in this context. However, this changed during a period at universities thanks to Ser Pilo Paga (SPP), a government program that granted forgivable loans to academically outstanding low-income students to study in high-quality universities in the country. The program lasted for about eight years, from 2015 when it started until 2023 when the majority of the last cohort graduated.

The SPP program highly increased the probability of high-achieving-low-income students to access high quality education (Álvarez Rivadulla et al., 2017; Londono-Velez et al., 2023). The entrance of these students to high quality universities, many of them elite institutions in the country, led to a change in the socio-demographic composition of the universities: for a moment, it was not only the privileged students from the capital city the ones attending the best Colombian universities, but also those coming from less privileged backgrounds and from all over the country. Nevertheless, this did not come without costs for low-income students. They had to adapt to social worlds that were different to theirs in many aspects, developing strategies like camouflaging, disclosure or omnivorousness to face relational costs and to interact with their higher-income peers and the university context in general (Álvarez–Rivadulla et al., 2023). Cross-class interactions also made low-income students more aware of inequalities and brought out what some have described as class wounds (Álvarez Rivadulla, 2014). These challenges related to social integration in elite university settings are common to the Latin American region, as showed by Quaresma and Villalobos (2022) regarding inequality reproduction in the Chilean case.

Although these impacts are relevant, less has been said about the effects of interactions on perceptions and attitudes toward inequality. The most important antecedent in the context of our study—and the only one to our knowledge— is the work by Londoño-Vélez (2022), which found that exposure to low-income peers has a modest though statistically significant effect in fostering a more accurate perception of inequality and more favorable attitudes toward redistribution among high-income students. The author suggests that the most privileged might become aware that there are more poor people than they initially thought or that they may recognize the merit of lower-income students, but she points out the difficulty of disentangling these mechanisms (Londoño-Vélez, 2022). As discussed below, our evidence supports these claims and clarifies how such mechanisms unfold.

## Data and methods

We primarily used a qualitative approach based on 61 in-depth interviews conducted at an elite college during 2016 until 2019, a period during which the diversity in this institution grew considerably. While in 2014, only 6,4% of the incoming students were from low socioeconomic status, in 2016, after the mentioned fellowship program to high achieving low-income students, that percentage grew to almost 25%. And low-income students kept entering this and other elite institutions with this program until 2019. We also used a network survey, with same cohort students of four different majors, to contextualize our findings (further information on the survey and the quantitative analysis can be found in the Supplementary Appendix). Both authors also conducted participant observations during the period of the study, one as a student and the other one as a professor at this elite institution, having the opportunity to engage in different informal conversations, class discussions and observe interactions as well.

We interviewed students from all social classes and from different majors at Study University. Table 1 shows further demographic information about these participants. Some of the interviews (34) were nested within the survey, selecting students according to their position in friendship networks and other relevant variables (e.g., if they were popular or isolated, from different social classes, and varying by gender). Other interviewees (27) were selected less systematically to gain a deeper understanding of the specific experiences of both upper and lower-class students. This less systematic sample aimed to search for greater variation and get a deeper comprehension of cross-class interactions. It also looked for evidence that corroborated or challenged the patterns we were identifying over the course of fieldwork. In other words, we relied on a qualitative sampling strategy based on quotas and inconvenient sampling (Small, 2009; Duneier, 2011). The interviews lasted between 1 and 2 h on average and discussed the students’ experience at Study University, their social networks, interactions with classmates from other backgrounds, perceptions of access to education in Colombia and opinions about SPP, besides information about their lives before entering the university as well to better contextualize their social positions and trajectories.

**Table 1:** In-depth interviews participants’ demographics.

Demographic characteristics		Interviews
Sex distribution		
	Men	38
	Women	23
Class distribution		
	Lower-class	44
	Middle-class	6
	Upper-class	11
Institution		
	Study university	56
	Other	5
Major		
	Social Sciences	11
	Psychology	7
	Medicine	16
	Business administration	5
	Law	2
	Economics	14
	Engineering	5
	Literature	1
Total *N* of interviews		61

Source: Authors’ elaboration based on interviews.

We analyzed interview transcriptions and field notes, focusing on identifying recurring themes related to students’ paths and experiences in college. The coding process combined both theoretical and inductive approaches, integrating categories coming from existing literature and allowing new ones to emerge. Some of our emergent codes were related to how students perceived inequality, meritocracy, social classes, the impact of diversity in university, and the SPP program. Theoretical codes included family and educational backgrounds, cultural capital and its subcategories, social capital and its subcategories, among others.

To divide students into social classes, we mixed two indicators that have been used in previous research about this topic and this case: (1) the division of Colombian households in six groups called “socioeconomic strata” (*estratos* in Spanish), which is based on an administrative classification of the affluence of the place of residence in cities according to which public services fees are calculated; and (2) whether students were beneficiaries of SPP or not. The first indicator is highly relevant since people in Colombia are more likely to identify with their *estrato* than with the class as traditionally defined. *Estrato* is deeply embedded in Colombian daily language as an indicator of social class (Uribe Mallarino and Pardo Perez, 2006) and correlates to education level and income. The second indicator, the categorical variable of being a SPP beneficiary or not, distinguishes lower-class students and is relevant to understand social relations in college (Álvarez–Rivadulla et al., 2023). Based on this, our social class division works as follows: all SPP students, as well as strata 1 and 2 students, were classified as lower-class; middle-class were those who came from socioeconomic strata 3 and 4 and were not SPP beneficiaries; finally, upper-class students were the ones who came from strata 5 and 6. This division was consistent with other socioeconomic measures: while upper-class students’ parents usually had professional or advanced university degrees, most lower-class ones only finished high school, and many times did not. The vast majority of lower-class students graduated from public schools—some of them outside Bogotá—while the upper-class ones came from private elite schools in Bogotá.

We use the terms lower-class student and scholarship student interchangeably in the text. All but one of the lower-class interviewees were scholarship recipients of SPP. Only one had a different fellowship because her father was a university worker.

Analytically, we focused on the following aspects to understand the relations among inequality perceptions, beliefs and distributive preferences: students’ perceptions of access to education in Colombia; their valuations and ideas of merit; own and others’ class experiences; and opinions about SPP. Given the educational segregation in the country, this issue elicited students’ perceptions of broader inequalities, as reflected in their experiences at school and university. Similarly, their ideas about merit revealed the extent to which this value was important in defining inequality. Perspectives on the SPP program were relevant because the program was understood as a redistributive policy that gave opportunities to those who deserved them and would not have had them otherwise due to material constraints.

## Results

**Figure 1:**
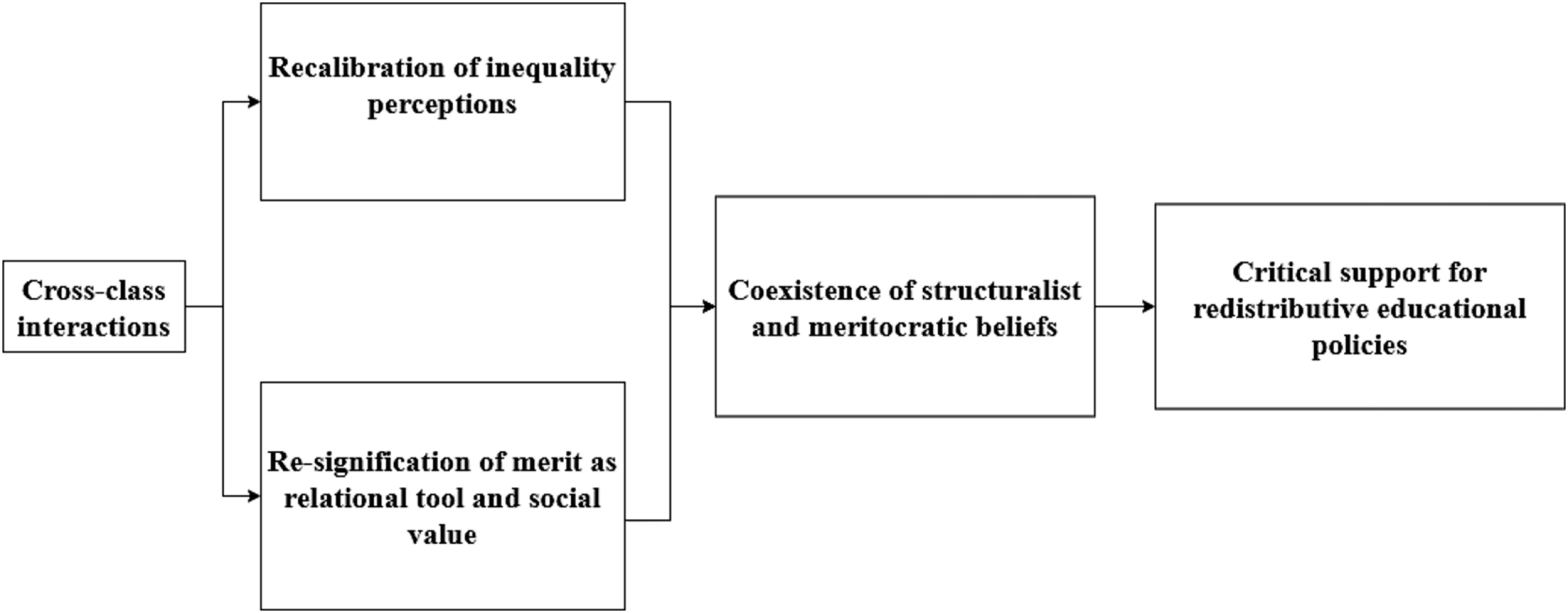
Results and conceptual relationships.

The following scheme summarizes our findings (see Figure 1), based on the mentioned conceptual developments and our empirical results on subjective inequality and cross-class interactions. Specifically, we contend that the policy-induced change in socioeconomic composition at Study University facilitated cross-class interactions that refined inequality perceptions through recalibration of inequality, as students from all social classes gained insights into the backgrounds and lived experiences of their peers. They also re-signified merit, understanding it not only as a value but also as a relational and integrational tool. However, students also questioned whether merit alone could overcome structural barriers. These changes in subjective comprehensions of inequality led to the coexistence of both structuralist and meritocratic views of inequality. Finally, this process influenced the formation of critical support regarding SPP as a redistributive policy in the domain of higher education: while many acknowledged its role in extending access to higher education, they also became aware of its limitations in addressing deeper structural inequalities. The next three subsections elaborate in greater detail these three main findings.

### Recalibrating inequality

Students from all social classes shared a structural view about socioeconomic inequality in Colombia and in education when we interviewed them. They all showed awareness about disparities in terms of income and opportunities that affected many in the country, saw it as a problem that did not depend only on individuals’ effort or talent but that was there as a contextual feature causing several problems. Yet, according to their accounts, this structural perspective became more refined thanks to contact with peers from diverse socioeconomic contexts. Through closer interactions with other socioeconomic groups that they had not had before, they all recalibrated inequality, understanding it was higher than they thought. While higher-income students learned from the experiences of their lower-income peers and got to know more concrete expressions of inequality than those they knew before, lower-income students became more aware of privilege and how distant their experiences growing up had been with respect to their upper-class peers.

The students’ structural understanding of inequality showed up when we talked with them about how easy they thought it was to enter to university in Colombia. None of them said it was easy for those with determination, grit, merit or effort. Upper class students tended to see inequality in education in terms of a public-private gap in academic quality. Emilio, a student that came from a top private school in Bogotá, told us the following:

It seems to me that getting into college is extremely difficult. I tell you this because our school, as I said, is the second-best school in the country. In terms of ICFES tests [local standardized state test], for instance, third best school. It's an incredibly good school. It is a very expensive one, too… And yet there were people in my graduating class who couldn't get into the universities they wanted to get into. This shows that even if you have all the possibilities and all the capabilities, getting into these universities is not easy at all. Now imagine being in a public school, where the teachers receive a miserable salary and they have to, clawing their way [con las uñas], try to figure out what to do and how to teach.

Lower-income students, in turn, displayed their structural view of inequality focusing on the economic barriers for accessing education. Since they overcame the hardships related to the quality of their public schools, their focus was on what they saw as an obstacle for those that, unlike them, could not attend university: money. One of our interviewees that received SPP told us that “[going to the university] is easy, it’s very reductionist, but it’s easy if you have the money. I mean, if you have enough money to pay your tuition.” Another lower-class student that applied to other universities before receiving the program puts it in these terms: “even though I had a very good ICFES score, I could not go to [another top university] because I did not have the money to pay tuition, I still do not have it.” Interestingly, although students from all social classes identified inequalities that limit access to education and they are concerned about it, they emphasized different topics, depending on their social position.

Both elite and lower-class students recalibrated their perception of inequality, making it more accurate, more vivid, more felt. Through interaction with low-income peers and knowing about their daily challenges, elite students became more aware of how life is for those who are not as privileged as them and, at the same time, more conscious of their own privilege. Melissa, an elite student, shared her reflections in the interview after talking about the experiences of some of her SPP classmates: “at school, I felt the way we lived was very normal. I sometimes even felt like lower class… But then you get here, and you realize that you are very privileged.” Pablo, another high-class student expressed a similar realization:

It's not the same knowing that you are going to study and then have lunch. [For scholarship students] it is ‘what am I going to have for lunch? how am I going to get by?’ That kind of thing is pretty hard.

This way, elite students recalibrated their inequality perceptions, gaining a deeper understanding of how their socioeconomic position contrasts with that of their less privileged peers.

Lower income students of course knew they were poor before entering into contact with more elite students. Yet, with contact, they realized what privilege really is and they saw a wider gap between what they have and have experienced, and what their classmates have and have experienced. This was especially so when they had closer encounters, such as when they visited their houses or when they spoke about their different recalls from their school environments or childhoods. It also became evident when they had to speak in English in a class and felt ashamed because some of their classmates had a native pronunciation, or when they went to lunch without caring about the costs and had to spend their weekly allowance on one day. Lina, a lower-income student expressed this in the following way:

One feels uncomfortable with another person who [hesitates], I don't know if you understand me. It's like they have everything, so maybe you feel a little bit overshadowed, a little bit different […] and you didn't have all those advantages, and you haven't traveled that much. Then comes the other person who has been to all the places, who has done a lot of things, who has had all the classes and who lives here in Bogotá with his family and does not live alone. So, it's not the same experience that one shares.

These perspectives illustrate how students from all social classes recalibrated their inequality perceptions thanks to their contact and interactions with individuals from other backgrounds. Becoming more aware of inequality for them entailed knowing different experiences that others had lived and understanding the place they occupied in an unequal society. For elite students, this generally implied getting to see the difficulties the lower-income ones lived with. For scholarship students, recalibrating inequality represented seeing in a, sometimes, harsher and closer way how far they were socially from their classmates in terms of the opportunities and resources they had up to that point in life. In the Supplementary Appendix, we present an exploratory analysis of survey data, showing that students across social classes reported greater support for redistribution by the state between 2017 to 2018. While we do not claim a causal link, this trend may stem from the recalibration of inequality perceptions and the strengthening of structural inequality beliefs.

### The meanings of merit

As mentioned before, literature often opposes a meritocratic perspective on inequality with a structural one. Yet, students in our sample held both. The emphasis on one or the other varied depending on social class. When speaking about their lower-class peers, upper class students tended to highlight their merit, that they were good students despite a lot of hardships and that they deserved to be in the university because of it. For the fellowship students, merit fostered recognition and validation from their upper-class peers. It was a source of pride and deservingness. Yet, they were more critical about merit than their upper-class peers, highlighting that merit is not enough to fully overcome structural barriers. They often mentioned people they knew, their siblings or people from their high schools who, despite comparable achievements to theirs, could not enter into a university.

Although elite students usually reported being unable to tell which of their classmates were SPP beneficiaries, they had a clear imaginary about how a scholarship student was. In general, they highlighted discipline and hard work as characteristics of these students. This was the backbone of the recognition and validation from elite students toward their SPP classmates. An elite student’s words illustrate this perception:

You get here and you see these guys, the few Pilos that I have met are the most responsible and hardest working people […] so, I think it's very brave and commendable. What they have done is very good, like [he applauds] [laughs]. Very good, really, for being able to stand out in a context that is designed for them not to stand out.

SPP recipients perceived this similarly, as they began to feel more acknowledged by their upper-class students when the latter recognized their merits and skills. Sergio, a lower-class student expresses it in the following way:

After the third semester onwards, when we began to define who was who academically, they [elite students] realized that one was not here just because, as if by chance. Relationships began to change […] They began to get closer to me, to talk, to ask me questions […] after we showed that we could really do things. Then the work groups were more diverse, I was already working with friends who were SPP and who were not. My best friend in college is not an SPP student.

At the same time, other scholarship students expressed that merit was not always enough to overcome structural barriers, because they saw this happen to some of their friends from school back at their places of origin. Maria, a lower-class student, told us the following about it:

In my class, there were girls who were very responsible and smart, but none of them got into a university. So, to see that there were people who wanted to, like them, who really wanted to get there and have a very good future and couldn't, that obviously makes me think that they deserved to be here. Of course, I am in the classes, and I think that they would be very happy here. It’s hard to see that they did not have that opportunity knowing that they had the talent.

These views show that merit acquires different meanings for each social class. For the upper-class students it is a motive to validate and recognize their low-income peers. For scholarship students, it is important but not always enough for overcoming barriers imposed by inequality. Nevertheless, there is a common understanding among all students of merit as a positive value, and this facilitates cross-class interactions at the university, turning merit into a relational tool. The Supplementary Appendix presents an exploration of our survey data, which shows stronger meritocratic beliefs in 2018 compared to 2017 across social classes. This may seem at odds with the structural awareness we documented earlier. However, this apparent contradiction can be understood through the coexisting views expressed in interviews: upper-class students came to recognize the difficulties faced by their lower-income peers, while simultaneously admiring their discipline and effort. Thus, greater exposure to SPP classmates may have reinforced these students’ appreciation for individual merit—even as they became aware of more structural inequalities. Rather than rejecting meritocracy, interactions appear to have strengthened a view of merit as morally valid and relational, even if not fully sufficient to overcome inequality.

### Critical support for Ser Pilo Paga

With a critical and structural perspective of inequality and such a high valuation of merit in this college context, we could expect a great support for a program such as the one that gave high achieving yet low-income individuals the possibility to enter the university of their choice. We should expect this, especially, among those more benefited by the policy. Yet, this was not always the case. In fact, a much more nuanced perspective of the program appeared and support for it diminished over time (see Supplementary Appendix).

Students showed opinions that identified both strengths and weaknesses of the SPP program. Those from the elite recognized that the program worked as an opportunity opener for many people that could not afford higher education by themselves. At the same time, the most privileged students saw as problematic that the program incentivized its recipients to attend private universities, taking away resources that could go to public ones. Additionally, interactions with lower-status peers made elite students aware of some hidden costs of the program, mostly related to adaptation to college life and social environment. On the low-income students’ side, they sometimes talked about the program as an opportunity opener as well, but they also identified the same flaws as the elite students, but also another one: the inclusion and exclusion errors of the program.

A lower-class student talked about opportunity opening when recalling a conversation she had with another scholarship recipient:

This program has really given the opportunity to many people to study. I was talking to another girl, and she said that she knew about SPP since ninth grade, and she prepared for three years to get it. I only got to know about it when I received my ICFES results [end of high-school state exam]! And she told me that she didn’t know what she would’ve done with her life without the scholarship.

Elite students showed very similar perceptions of the impact that SPP had on the lives of the beneficiaries as an opener of previously closed opportunities.

However, students from all social classes recognized that the program took most of scholarship students to private universities, thus using public resources in these institutions instead of public ones, which are usually more accessible for the underprivileged. They saw this as contributing to the critical financial situation that public universities face in Colombia. This was problematic for them, regardless of class position. Emilio, the elite student we mentioned before, said that:

it is a very large investment that should be better planned to strengthen public education in the country. […] Investing this money in strengthening public education, seems to me that it would be much better than what is being done now with Ser Pilo Paga.

Valentina, a lower-class student also identified this issue: “I think this is going to sound contradictory because I am from the program, but, I mean, we must be critical. The money that is being allocated for SPP is going at a much higher percentage to private universities.” Thus, students from different classes criticized it specifically regarding the disproportionate use of public resources in private schools that, for them, could be better invested in public universities and give these opportunities to larger segments of lower-class students.

Another critique identified, particularly among lower-class students, was the existence of errors of inclusion and exclusion in the program. Camila, a scholarship student told us about a friend of hers that received SPP but did not need it, according to her:

There are people who have the money to study in good universities. To at least study. Let's say I was talking to a friend of mine, and she is SPP, and she told me ‘My parents have the money to pay my tuition at [another top university] but not the tuition here at Study University’. So, I thought she has the resources and meanwhile there are people who really have nothing.

Valentina also saw freeloaders (*colados* in Spanish) as a problem, and like many others, had a negative moral boundary set against them, based on taking opportunities from others.

There are colados, yes, [laughs] and that’s really wrong. For example, some of my high school classmates reached the ICFES score [state exam score], but not the SISBEN score [poverty score]. And, if you compare, they deserved the scholarship more than many colados.^2^

This aspect nuances the perceptions of the SPP program especially from the point of view of those who directly benefit from it and brings nuances to ideas of deservingness in such an unequal society. These views reflect what we refer to as *critical support* for the program: students acknowledged the opportunities SPP created, especially for talented students with low resources, but they also became aware of its limitations. Importantly, critique did not necessarily dampen support. Rather, it often coexisted with an appreciation for the program’s redistributive objective.

In the Supplementary Appendix, we present an analysis exploring variation in support for the SPP program, which shows a decline over time across the sample. This pattern aligns with the more critical views documented in the interviews. However, qualitative data also revealed widespread recognition of the program’s positive effects, suggesting that support did not simply diminish, but rather became more nuanced among students from all social classes.

## Discussion and conclusion

Students from all social classes held structural inequality beliefs, this is, a view that inequality was a contextual problem in Colombia affecting the lives of individuals regardless of their effort and talent. This perspective was evident when asked directly how difficult it was to enter university in their country, and they immediately mentioned socioeconomic and academic barriers. We did not find anybody who said phrases such as “if I did, everybody can,” or “with effort, you can enter,” which we could expect, based on the self-interest hypothesis, either from those moving up in the social ladder wanting to underline their exceptionality, or from those that being up do not see inequality as a problem because it has not affected them.

As students interact with peers from different socioeconomic contexts, they develop more accurate views of inequality and, in turn, place greater importance on redistribution to reduce such inequalities. By becoming more conscious of poverty and its restraints and privilege and its benefits, students of all social classes become more aware of the external constraints that inequality puts on less privileged individuals, and prompt to the idea that somethings needs to be done to better redistribute opportunities, linking structural beliefs of inequality to lower tolerance toward it. Thus, our findings are consistent with previous literature that suggests that contact and cross-class interactions promote more accurate perceptions of inequality and more redistributive preferences (Newman, 2014; Rao, 2019; Londoño-Vélez, 2022; Sumaktoyo et al., 2022; Otero and Mendoza, 2023). In other words, the findings in our study appear to be consistent with Allport’s contact hypothesis (Allport, 1954), as cross-class contact seemed to contribute to shifts in students’ views of other social classes and inequalities overall, thanks to the recalibration of inequality that reduced prejudices and made them more aware of the other’s situations, especially that of the more disadvantaged.

The second big finding of this research regards the relationship between this structural perspective and a strong belief in effort and merit. Effort, merit, and meritocracy tend to be associated with more individualistic perspectives about inequality. Yet, among the students of this study, a strong valuation of effort and merit coexisted with a structural perspective, in a similar fashion to that of the split consciousness (Castillo, 2011), but this also led to more cross-class interactions which in turn led to an even more structuralist perspective. Qualitative analysis of the narratives gave us the tools to understand the relevance of merit, and in particular the relevance of merit to promote rather than erode more egalitarian distributive attitudes in this particular educational context. Contrary to the self-interest hypothesis (Meltzer and Richard, 1981), students of all social classes valued merit as the basis for equality in a society so inegalitarian that your birthplace and your mother’s education often determines your educational future and life chances (García et al., 2015). For the upper-class students, merit was the basis for respecting and admiring fellowship students as well as for questioning their own privileged trajectories. For the lower-class students, merit was the basis for pride and deservingness. Yet, contrary to the self-interest hypothesis again, they were not legitimizing inequality based on merit. They did not feel, in general, that they deserved the fellowship more than others they knew from school, family or community. They felt luck played a role in their trajectories and that kept them conscious of the structural causes of inequality. Meritocratic beliefs are indeed important for understanding how people interpret inequality in their daily lives, as social sciences literature has shown (Mijs, 2021). However, our findings show that meritocracy not only works as an idea that legitimizes inequality, but it also may be an instrument for promoting not only more egalitarian views but also cross-class interactions.

The interviews also helped us understand one of the most puzzling results: criticisms of the SPP program (especially puzzling when considered in isolation in the survey, as seen in the Supplementary Appendix, less so when we analyze it as a narrative in relation to other attitudes toward inequality). Interestingly, this agrees with rather than contradicts a growing structural perspective on inequality. With the fellowship, discussions about educational inequalities became a thematized issue in elite universities. The national discussions on educational disparities between public and private education entered the gates of elite institutions. The left and the student movement opposed SPP because it was transferring public resources to private universities rather than only spending in public institutions. In many ways, students of different social classes saw SPP as not structural enough. Criticism to inclusion and exclusion mistakes in assigning the fellowship might have played a role in the erosion of support for SPP as a redistributive mechanism. Yet, the relevant consideration here is that this diminishing support for this fellowship coexisted rather than contradicted with a growing awareness and preoccupation for the educational inequalities of the country.

These findings are grounded in rich qualitative data, complemented by secondary survey data exploratory analysis that contributed to contextualizing and nuancing our findings. This combination highlights the value of mixed-methods approaches in studying subjective inequality. It also raises further questions about how cross-class interactions shape attitudinal change, underscoring the need for future research that builds on the depth of qualitative data while incorporating quantitative designs focused on testing causal mechanisms and relationships, to better contextualize and explain inequality perceptions and beliefs.

Although SPP was not intentionally designed to promote cross-class interactions, this was a result of socioeconomic diversity induced by increased access of lower-class students to elite universities. These changes also contributed to the shifts in students’ attitudes toward inequality we analyzed here. This points to relevant policy implications: promoting contact among students from different socioeconomic backgrounds may rise awareness regarding inequality and simultaneously generate critical reflections on redistribution—not because of a rejection of redistribution itself, but because students come to recognize its scope, limits and implementation flaws.

While our findings are grounded in a specific policy and institutional setting, the relational mechanisms of subjective inequality change outlined in this study—like the recalibration of inequality perceptions, the resignification of merit and the critical support of redistributive policies—may provide useful insights for analyzing other cases of cross-class interactions in segregated and highly unequal contexts. These mechanisms can be particularly relevant in cases where class mixing occurs in spaces or contexts traditionally occupied by the most privileged, and where exposure to inequality is experienced through everyday interactions in meaningful and socializing spaces, such as educational settings.

In sum, our research contributes in at least three ways to the literature on subjective inequality and distributive attitudes. First, we connect them with literature on contact and prejudice, and we argue that cross-class interactions may promote more distributive attitudes based on more accurate assessments of social distances and inequality levels. Second, we show that meritocratic beliefs do not often act legitimizing inequality but sometimes promoting more egalitarian views. This is based on merit as a promoter of interactions among different social classes in contexts that value merit, such as educational ones. Finally, our findings confirm the multidimensionality and complexity of distributive preferences highlighted by the literature (Cavaillé and Trump, 2015) underlying the role of qualitative methods or mixed methods to delve deeper into the apparent contradictions among different attitudes, looking at their narrative connections and paying attention to the relational contexts in which those attitudes emerge.

## Data Availability

The raw data supporting the conclusions of this article will be made available by the authors, without undue reservation.

## Footnotes

^1^For exceptions see the work by Stuber (2006) and Mijs (2023).

^2^The two most important conditions to be part of the program were passing a threshold of the ICFES score and being in a certain range of SISBEN scores. SISBEN is a Colombian system that classifies people in levels of vulnerability and poverty according to different socioeconomic characteristics of households. It is used to target different types of social programs.
