# Supplementary material for: Cross-class interactions and subjective inequality: perceptions, beliefs and distributive preferences at a Colombian elite university
Source: Front Sociol. 2025 Sep 16;10:1619937. doi: 10.3389/fsoc.2025.1619937 (PMC12481291; doi:10.3389/fsoc.2025.1619937)
Supplement: Supplementary file 1 [file Presentation_1.pdf]

**Supplementary Appendix for Cross-class interactions and subjective inequality: perceptions, beliefs and distributive preferences at a Colombian elite university**

## Exploratory analysis of survey data

The survey was administered to students majoring in four disciplines from different knowledge areas at an elite college in Colombia dubbed Study University, and with different class compositions (a differential proportion of lower-, middle- and upper-class students giving different structural opportunities for cross-class interaction). The first wave of the survey was applied in 2017 and once again, to the same students, in 2018 to look for changes over time. We sent an email to all the students of these four disciplines inviting them to participate with the only incentive of a raffle of gift cards for a famous burger chain. The response rate was good (between 82% of and 50% in the first wave and between 67% and 54% in the second wave) and the demographic analysis in terms of gender, Grade Point Average (GPA), and social class was like that of their respective cohorts (see Table 2). 149 students completed the survey in both waves, but we did not exclude those who did not complete it because we had information about them through the University Registrar's Office and networks data from what other students reported in the survey. The questionnaire included sociodemographic characterization questions that students typically answer for the University Registrar's Office, as well as some specific questions that sought to assess students' social capital and diversity in social networks, their subjective social class and mobility expectations, and their perceptions of inequality and distributive preferences.

**Table A1. Survey respondents' demographics and comparison to their cohorts**

| Category | | Respondents | | 2017 Cohort | |
|---|---|---|---|---|---|
| | | *N* | *%* | *N* | *%* |
| **Major** | | | | | |
| | Major 1 | 51 | 34.23 | 76 | 31.15 |
| | Major 2 | 38 | 25.50 | 70 | 28.69 |
| | Major 3 | 36 | 24.16 | 56 | 22.95 |
| | Major 4 | 24 | 16.11 | 42 | 17.21 |
| **Gender distribution** | | | | | |
| | Male | 90 | 60.40 | 148 | 60.66 |
| | Female | 59 | 39.60 | 93 | 38.11 |
| **Scholarship status distribution** | | | | | |
| | Ser Pilo Paga scholarship holder | 93 | 62.42 | 143 | 58.61 |
| | Non-Ser Pilo Paga scholarship holder | 56 | 37.58 | 92 | 37.70 |
| **SES distribution** | | | | | |
| | Low-SES | 58 | 38.93% | 98 | 40.16% |
| | Middle-SES | 52 | 34.90% | 73 | 29.92% |
| | High-SES | 38 | 25.50% | 61 | 25.00% |

| 2018–2 GPA (mean) | Mean | Std. dev | Mean | Std. dev |
|---|---|---|---|---|
| GPA | 3.87 | 0.35 | 3.78 | 0.44 |
| **Total** | 149 | | 244 | |

**Source:** Authors based on Networks Survey and Study University Registrar's Office data

We analyzed how perceptions of inequality and distributive preferences changed from 2017 to 2018 among students from all social classes. We focused on their preference of income redistribution by the government, their evaluation of perceived benefits of inequality, and their attitudes towards the continuation of the SPP program. To divide students into social classes, we mixed two indicators: socioeconomic strata, which is a division of Colombian households in six groups based on place of residence, and whether students were beneficiaries of SPP or not. Thus, we considered lower-class all SPP recipients and students from strata 1 and 2 that did not have the scholarship; middle-class included students from strata 3 and 4; and elite students were those from strata 5 and 6. This section presents the results of this analysis in descriptive terms, showing the percentage of students by social class who answered "Completely agree" or "Agree" to each one of the survey items in the following figures:

Figure A1.

Figure A1 here

**Source:** Authors based on Networks Survey

Figure A1 shows the change in distributive preferences from one year to the next for the upper, middle and lower classes. The question measuring preferences here is the classic survey question for operationalizing redistributive demands: "How much do you agree with the statement 'The state should implement firm policies to reduce inequality among rich and poor'?" In this regard we can say that in 2018 the number of students who have more favorable preferences towards redistribution is higher than in 2017. In all three social classes, the percentage of students in the "agree" category in 2018 almost doubles the quantity of 2017. This may imply a positive change in support for redistribution from one year to the next that does not seem to depend on socioeconomic status. However, as our qualitative results showed, inequality awareness and redistributive support were already extended among students in the first period, which is also interesting.

Figure A2.

Figure A2 here

**Source:** Authors based on Networks Survey

In this second graph, we observe a measure of inequality beliefs. Figure A2 examines the extent to which inequality is accepted based on the perceived benefit it has for poorer individuals, as reflected in the survey statement that inequality motivates them to try harder.

This is associated with valuations of effort, and more broadly to meritocratic beliefs. Once again, agreement with the statement grows among students of all social classes. This result, a very functionalist perspective and apparently contradictory with the more structural perspective on inequality, was puzzling at the beginning. Yet, qualitative data gave us the tools to understand the relevance of merit, and in particular the relevance of merit to promote rather than erode more egalitarian distributive attitudes in this particular educational context, via enabling cross-class interactions.

Figure A3.

Figure A3 here

**Source:** Authors based on Networks Survey

Figure A3 shows how support for the SPP program changes over time. This is one of the most surprising trends in our data, as support declines over time for all social classes. In 2017, all three classes showed most responses in the "agree" category. By 2018, although the lower class is the group most in favor of continuing SPP, the share of students supporting the program also decreased. As showed in the article, qualitative data gave us tools to understand this more critical perspective towards the program.

In sum, the survey was useful to generate questions and to further explore inequality beliefs and distributive attitudes at Study University in the initial years of our study. Although the richness of our data is in the qualitative data presented in the core of the article, we wanted to show the interested reader the power of mixed methods to better understand the complexities of distributive attitudes, their apparent contradictions and their profound relational nature. Without situating distributive attitudes in specific contexts, with different degrees of objective inequality and segregation they become difficult to interpret on their own.
